# Combined Therapy of Hypertensive Nephropathy with Breviscapine Injection and Antihypertensive Drugs: A Systematic Review and a Meta-Analysis

**DOI:** 10.1155/2018/2958717

**Published:** 2018-12-20

**Authors:** Lihua Wu, Ming Liu, Zhuyuan Fang

**Affiliations:** ^1^Affiliated Hospital of Nanjing University of Chinese Medicine, Nanjing, Jiangsu 210029, China; ^2^Institute of Hypertension, Affiliated Hospital of Nanjing University of Chinese Medicine, Nanjing, Jiangsu 210029, China

## Abstract

**Objective:**

To evaluate the beneficial and adverse effects of breviscapine injection in combination with antihypertensive drugs for treating hypertensive nephropathy in clinical practice.

**Methods:**

We searched PubMed, the Cochrane Library, Embase, CNKI, Sino Med, VIP, and Wanfang Data for relevant literature. The timeframe of retrieval was set from the founding date of each database to September 28, 2018.

**Results:**

Fourteen papers were included in this study. The quality of all the studies included was determined to be low. All studies were conducted with Chinese populations. Meta-analysis showed that, compared with single-use antihypertensive drugs, using breviscapine injection in combination with antihypertensive drugs to treat hypertensive nephropathy can reduce serum creatinine (Scr) [WMD = –35.16, 95% CI(–50.01, –20.31), *P* ≤ 0.001], blood urea nitrogen (BUN) [WMD = –2.00, 95% CI(–3.07, –0.94), *P* ≤ 0.001], 24-hour urinary total protein (24 h UTP) [WMD = –0.04, 95% CI(–0.05, –0.02), *P* ≤ 0.001], and the beta-2-microglobulin (B2M) [WMD = –0.09, 95% CI(–0.11, –0.07), *P* ≤ 0.001], improve creatinine clearance rate (Ccr) [WMD = 7.84, 95% CI(5.20, 10.49), *P* ≤ 0.001], and increase the clinical efficacy [RR = 1.27, 95% CI(1.05, 1.53), *P* = 0.014], but does not lower systolic blood pressure (SBP) [WMD = –1.02, 95% CI(–2.88, 0.84), *P* = 0.281]. There was no significant difference in adverse events between experimental groups and control groups.

**Conclusion:**

Breviscapine injection in combination with antihypertensive drugs can improve clinical efficacy and Ccr and reduce Scr, BUN, 24 h UTP, and B2M in patients with hypertensive nephropathy. The present meta-analysis indicated that breviscapine injection can serve as a renal protective effect to patients with hypertensive nephropathy. However, the evidence of methodological quality and sample sizes is weak, and thus, further standardized research is required.

## 1. Introduction

Hypertension is a risk factor for stroke, coronary artery disease, heart failure, peripheral vascular disease, and chronic kidney disease (CKD) [1, 2]. Elevated blood pressure (BP) was the leading global contributor to premature death in 2015, accounting for almost 10 million deaths and over 200 million disability-adjusted life years [2]. The main factors leading to the development of hypertensive nephropathy include (1) inappropriately elevated sympathetic nervous activity (SNA) [3]; (2) activation of the renin-angiotensin-aldosterone system (RAAS) [4]; (3) increased arterial stiffness [5]; (4) genetic susceptibility [6]; (5) impaired salt and water excretion by the kidney [7]. Hypertensive kidney disease is the second leading cause of end-stage renal disease (ESRD) after diabetes mellitus [8, 9]. In Europe, according to the European Dialysis and Transplant Association registry, hypertensive nephropathy is accounted for 12% of new patients starting renal replacement therapy. However, the reported incidence varies among different countries, with France, Italy, and United Kingdom, reporting in 25%, 17%, and 6.1%, with both Japanese and Chinese reporting in 6% and 7%, respectively [10]. Chronic kidney failure is a global disease: at the end of 2016, approximately 3 million patients were on dialysis. The incidence of chronic kidney failure varies between regions, North America 0.638 million, Europe, Middle East, and Africa 0.711 million, Asia-Pacific 1.343 million, and Latin America 0.288 million [11]. The diagnosis of hypertension-induced renal damage is based on the finding of reduced renal function and/or the detection of albuminuria (≥300mg/d, or ≥300mg/g albuminuria to-creatinine ratio in the first morning void). CKD is classified according to estimated glomerular filtration rate (eGFR), calculated by the 2009 CKD-Epidemiology Collaboration formula [12]. Current evidence suggests that, in patients with CKD, BP should be lowered to less than 140/90 mmHg and towards 130/80 mmHg. The combination of renin-angiotensin system blockers with calcium channel blockers (CCB) or diuretics should be used to achieve recommended blood pressure targets in CKD [9, 13]. A recent meta-analysis has shown that BP lowering significantly reduced ESRD in patients with CKD, but only in patients with albuminuria, and had no beneficial effect on cardiovascular events [14]. In a large retrospective cohort containing 398419 treated hypertensive patients, the nadir systolic blood pressure (SBP) and diastolic blood pressure (DBP) for the lowest risk of ESRD and mortality were 137 and 71 mmHg, respectively, with a clear increase in mortality risk at SBP less than 120 mmHg [15]. The evidence with respect to BP targets in patients with CKD is complex. Lowering blood pressure may lead to a decrease in eGFR. The reduction of albuminuria is also considered to be a therapeutic target. However, there are also studies in which treatment that was less effective at reducing albuminuria was more effective at reducing cardiovascular events [16].

Breviscapine (Dengzhanhua) injection is extracted from Erigeron breviscapus (Vant.), Erigeron breviscapus also known as herba erigerontis or lamp chrysanthemum, is a traditional Chinese herb that has been in use for more than 600 years, found in Yunnan, Sichuan, Guizhou, and other southwest provinces of China. Breviscapine, as a purified flavonoid extract from this species, was first isolated by Zhang et al. [17]. Breviscapine mainly contains scutellarin (4′,5,6,7-tetrahydroxyflavone-7-O-glucuronide) and apigenin-7-O-glucuronide [18]. Studies have shown that breviscapine has significant effects on vasodilation; inhibition of platelet aggregation, scavenging free radicals, also has a protective effect on myocardial and endothelial structures because of its anti-inflammatory effects and improves microcirculation; protection against ischemia/reperfusion (I/R); anticoagulation and antithrombosis; reduction of smooth muscle cell migration and proliferation; anticardiac remodeling; antiarrhythmia; and reduction of blood lipids [19–22]. Breviscapine has been demonstrated to possess a number of pharmacological functions in addition to its hemodynamic effects; it has been reported to serve as an antioxidative stress agent and a protein kinase C (PKC) inhibitor, can inhibit the glycogen synthase kinase 3*β* (GSK3*β*) signaling pathway to promote neurobehavioral function following neurotrauma, and can improve renal function and reduce urinary microalbuminuria [23–26]. In the light of these pharmacological activities, an injection preparation of breviscapine (a traditional Chinese patent medicine) has been wildly used in clinical treatment for cerebral infarction, cardiovascular disease, diabetic nephropathy, renal impairment of essential hypertension, and stroke in China [18, 25, 27, 28].

However, in the past decades, although numerous studies have compared breviscapine injection with antihypertensive drugs in the treatment of hypertensive nephropathy, the comparability of treatment protocols and evaluation methodologies among these studies remains to be proven, which greatly limits their clinical applicability. Furthermore, the current state of evidence of breviscapine injection for hypertensive nephropathy has so far been unknown. Therefore, we conducted this systematic review to evaluate beneficial and adverse effects of breviscapine injection in the treatment of hypertensive nephropathy.

## 2. Methods and Analysis

### 2.1. Search Strategy

We designed our systematic review and meta-analysis in accordance with the guidelines of the 2009 Preferred Reporting Items for Systematic Reviews and Meta-analysis (PRISMA) statement. Electronic network databases were searched via computer. Foreign databases searched included PubMed, Embase, and the Cochrane Library. Chinese databases included the China National Knowledge Infrastructure (CNKI), China Biology Medicine Disc (Sino Med), the VIP information resource integration service platform (VIP), and the Wanfang Data knowledge service platform (Wanfang Data). The retrieval scheme was mainly based on a combination of subject words and free words. The searched words were “Dengzhanhua”, “Dengzhanhua preparations”, “Dengzhanhua Zhusheye”, “Zhusheyong Dengzhanhua”, “Gaoxueya Shenbing”, “Gaoxueya Shenyan”, “Gaoxueya Shenshunhai”, while the searched English words were “Breviscapine”, “Breviscapine Injection”, “BVP”, “Hypertensive Nephropathy”, “Hypertension, Renal”, “Hypertensive Kidney Lesion”, “Hypertensive Renal Damage” and so on (Supplementary Tables S1 and S2 for the search strategy). The retrieval language was not limited, and the timeframe of the retrieval was from the founding date of each database to September 28, 2018. There was no language limitation. Manual searches of relevant literature supplemented the electronic search.

### 2.2. Inclusion and Exclusion Criteria

#### 2.2.1. Types of Studies

Randomized controlled trials (RCTs) that use breviscapine injection in combination with antihypertensive drugs to treat hypertensive nephropathy, regardless of blinding, were used in this study. Language was not restricted as to minimize publication bias.

#### 2.2.2. Types of Participants

There were no serious organic diseases or complications in the selected cases. The diagnosis of hypertension-induced renal damage is based on the finding of reduced renal function and/or the detection of albuminuria. CKD is classified according to eGFR, calculated by the 2009 CKD-Epidemiologyn Collaboration formula [12]. Hypertension was defined as SBP ≥ 140 mmHg or DBP ≥ 90 mmHg based on the Chinese Guidelines for the Prevention and Treatment of Hypertension (2010) and the 8th session of the Committee, Report of the Joint Commission/JNC8; secondary hypertension and primary heart, liver, and brain disorder were excluded. We did not limit inclusion based on age, sex, case source, disease course, hypertensive classification, or antihypertensives.

#### 2.2.3. Exclusion of Studies

Studies were excluded if they were (1) clinical trials from which no relevant data could be extracted; (2) studies that were published repeatedly; (3) populated with inconsistent baseline information (age, sex, case source, disease course, hypertensive classification, or antihypertensive drugs); (4) systematic review, important data report, and case reports; no reply from corresponding authors such that further data could not be obtained; and (5) therapeutic measures failing to meet the predetermined inclusion criteria.

#### 2.2.4. Intervention

Experimental group received breviscapine injection combined with antihypertensive drugs [captopril, amlodipine, lisinopril, benazepril, losartan potassium, felodipine, and nifedipine (medication dose, medication time and frequency, and treatment course)]. Control group received antihypertensive drugs including captopril, amlodipine, lisinopril, benazepril, losartan potassium, felodipine, nifedipine (medication dose, medication time and frequency, and treatment course). Age, sex, and other baseline conditions of research subjects were well matched.

#### 2.2.5. Types of Outcome Measures

Primary outcomes were serum creatinine (Scr) and 24-hour urinary total protein (24 h UTP). Secondary outcomes included blood urea nitrogen (BUN), creatinine clearance rate (Ccr), beta-2-microglobulin (B2M), systolic blood pressure (SBP), clinical efficacy, and adverse effects.

#### 2.2.6. Data Extraction

Two evaluators independently performed a search according to the search strategy, and preliminary screening was based on independent topics and abstracts of the search results, excluding obviously unqualified documents. A full-text methodology screening was conducted on the literature that might meet the inclusion criteria, and the authors were contacted when there was incomplete information. Then, the studies were cross-checked by two evaluators. Any disagreement on the conclusion of two evaluators was resolved by discussion. If such disagreement could not be resolved through discussion, final judgment and arbitration were made by a third party. Extracted contents included authors' names, year of publication, number of samples, intervention, course of treatment, and observed indicators.

### 2.3. Quality Evaluation

The investigators simultaneously evaluated the bias risk of the included studies based on the “risk of bias” evaluation tool in the Cochrane Handbook for Systematic Reviews [42] of interventions and relevant assessment guideline regulations. This risk-evaluation tool contains seven items, (1) random sequence generation; (2) allocation concealment; (3) blinding of the participants and personnel; (4) blinding of the outcome data; (5) incomplete outcome data; (6) selective reporting; and (7) other bias, and were evaluated as having a “high risk of bias”, “low risk of bias”, or “unclear risk of bias” according to assessment criteria.

### 2.4. Data Analysis

(1) Stata 14.0 software was used to perform the statistical analysis for the meta-analysis [43]. (2) Select effect size: if an index of the included documents is a binary variable, the curative effect analysis statistics can be represented by relative risk (RR) and expressed by its confidence interval (CI); mean difference (MD) and 95% CI were used to represent continuous changes. (3) Homogeneity test: it tests the variation degree of original research results and clearly includes the degree of homogeneity of the experiment. (4) Meta-analysis: according to the result of the heterogeneity test,* P *≥ 0.05 and *I*^2^ < 50 indicate that the results have good agreement and that the fixed effect model may be used. *P* < 0.05 and *I*^2^ ≥ 50 suggest that the heterogeneity of the results cannot be ignored. If the included studies still have clinical significance, the random effects model may be used. (5) Sensitivity analysis: in those meta-analyses of the comprehensive factors combined with multiple outcomes, possible anomalous studies were ruled out before reevaluation. The results were compared with those of meta-analysis before the exclusion to determine how the excluded studies would influence the combined effect size and the stability of meta-analysis. If there is little difference between the two results, then the sensitivity of the results is relatively low, and the results are stable, indicating high credibility. (6) Subgroup analysis: subgroup analysis was conducted on some indexes with high heterogeneity. For events in which quantitative synthesis was impossible and events with very low incidence, qualitative evaluation may be based on the description. In this study, Stata 14.0 software was used to conduct a sensitivity analysis and subgroup analysis and to create a sensitivity analysis chart.

### 2.5. Publication Bias

Publication bias occurs when positive data in similar research papers with statistical significance are more likely to be published in journals. This situation is hard to control. The funnel plot method is often used to detect publication bias. Egger's test was performed to detect publication bias in the outcome measures. If a large publication bias was found in a particular research index, the exact reason was identified.

## 3. Results

### 3.1. Search Results

**Figure 1:**
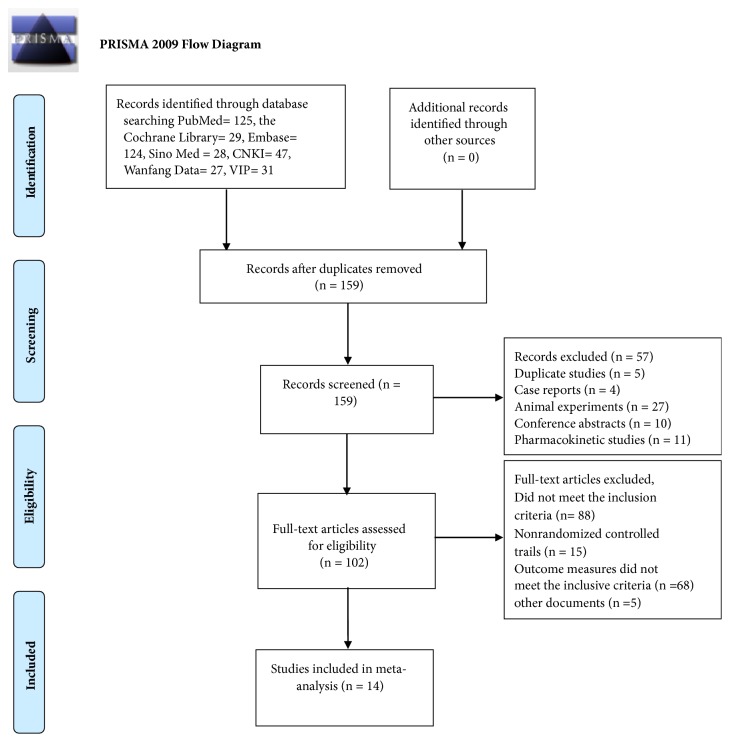
Flowchart of the process for literature retrieval.

A total of 411 documents [PubMed (n = 125), the Cochrane Library (n= 29), Embase (n = 124), Sino Med (n = 28), CNKI (n = 47), Wanfang Data (n =27), and VIP (n = 31)] met the data collection and search strategy conditions. NoteExpress, a professional document management software, was employed to check for duplication of the 411 obtained articles that met the relevance requirement. The majority of these trials were excluded because some papers were found in more than one database and some included irrelevant titles and abstracts. Only 159 studies were retrieved. Following a review of the titles and abstracts, several studies were excluded, and only 102 studies remained. Five trials were excluded because of duplicated publications. Twenty-seven trials were excluded for being animal studies, and twenty-five trials were excluded for being nonclinical trials, including case reports, pharmacokinetic studies, and conference abstracts. Eighty-eight out of the remaining 102 articles were excluded based on the inclusion criteria, leaving fourteen RCTs to be reviewed in Figure 1.

### 3.2. Study Characteristics

There were 14 randomized controlled trials [29–41, 44] that were included in the present research involving 1,170 patients (593 in the research group and 577 in the control group). These 14 RCTs are summarized in Table 1.

### 3.3. Summary of the Quality and Bias Risk of the Trials Included

**Figure 2:**
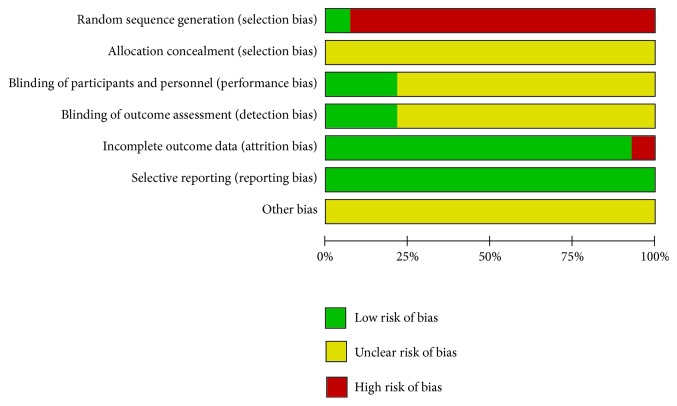
Risk of bias.

**Figure 3:**
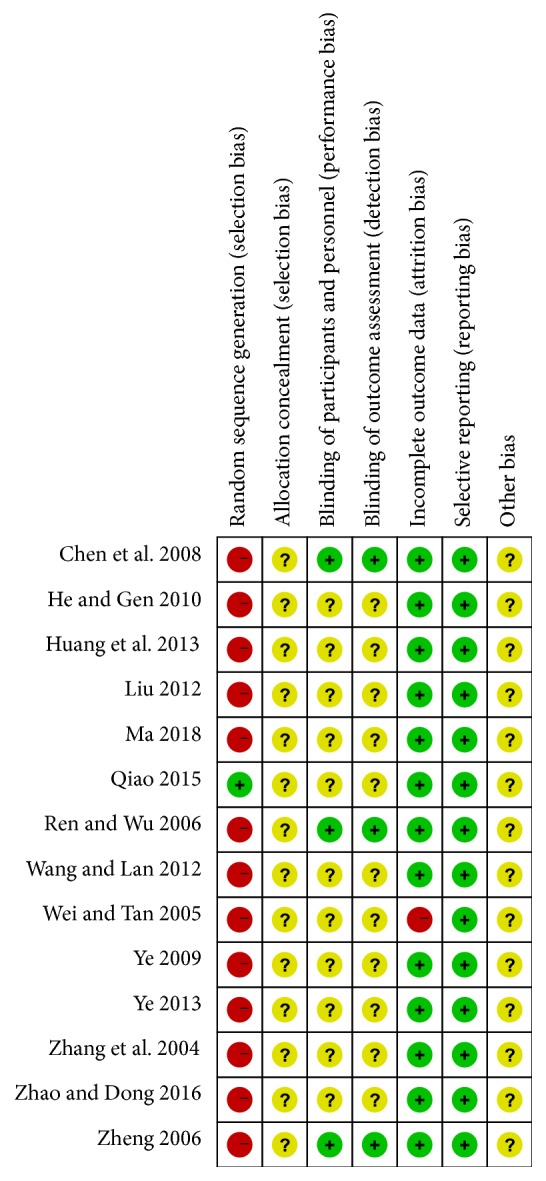
Risk of bias summary and graph.

The quality of all studies included was low. All studies were carried out among the Chinese population. Fourteen studies mention the use of random allocation: All studies failed to mention the specific grouping method, and none of the studies discussed allocation concealment, blinding, or evaluator blinding. The quality assessment is shown in Figures 2 and 3.

### 3.4. Outcome Measures

#### 3.4.1. Primary Outcome: Serum Creatinine (Scr, *μ*mol/l)

**Figure 4:**
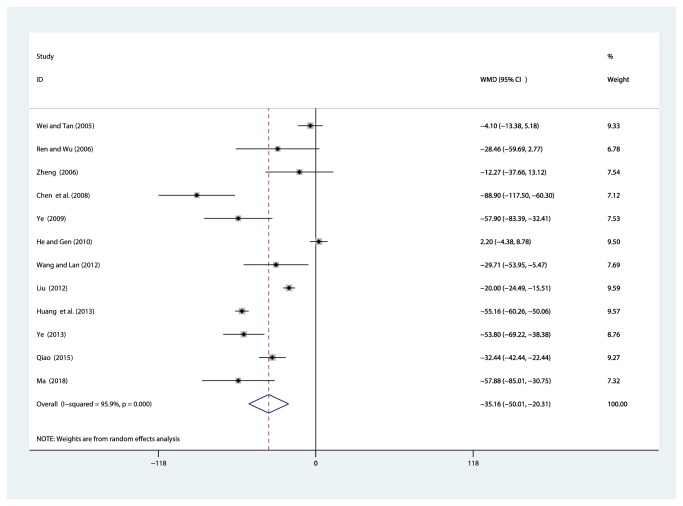
Meta-analysis results of breviscapine injection plus antihypertensive drugs versus antihypertensive drugs alone in terms of the Scr for hypertensive nephropathy.

**Figure 5:**
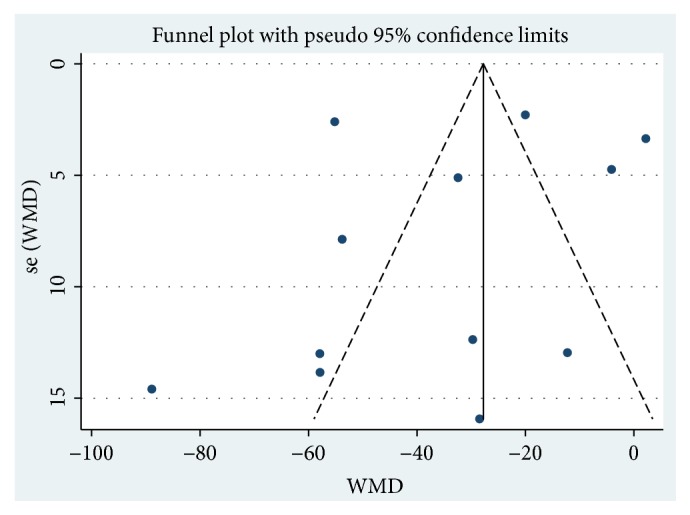
Funnel plot for the publication bias of Scr.

Twelve studies [30–40, 44] involving 969 participants reported on the use of breviscapine injection plus antihypertensive drugs in the treatment of Scr for hypertensive nephropathy. After the test for heterogeneity (*I*^2^ = 95.9%, *P* ≤ 0.001) (Figure 4), we employed a random effects model. A funnel plot analysis of the 12 trials suggested possible publication bias and inclusion of low quality studies as significant asymmetry is shown in Figure 5. We applied Egger's test to evaluate publication bias. A p (*P *= 0.634) value more than 0.05 was considered no publication bias (Supplementary Figure S1). The meta-analysis revealed that the experimental group performed better than the control group in reducing Scr [WMD = –35.16, 95% CI(–50.01, –20.31), *P* ≤ 0.001] (Supplementary Figure S2).

#### 3.4.2. Sensitivity Analysis

We conducted a sensitivity analysis for Scr (Supplementary Figure S3). By seriatim excluding one trial each time and reperforming meta-analysis of the remaining trials, we could observe whether the outcomes have dramatically changed. Sensitivity analysis indicated that the outcomes of Scr were very similar, which had relatively good stability.

#### 3.4.3. Subgroup Analysis

**Figure 6:**
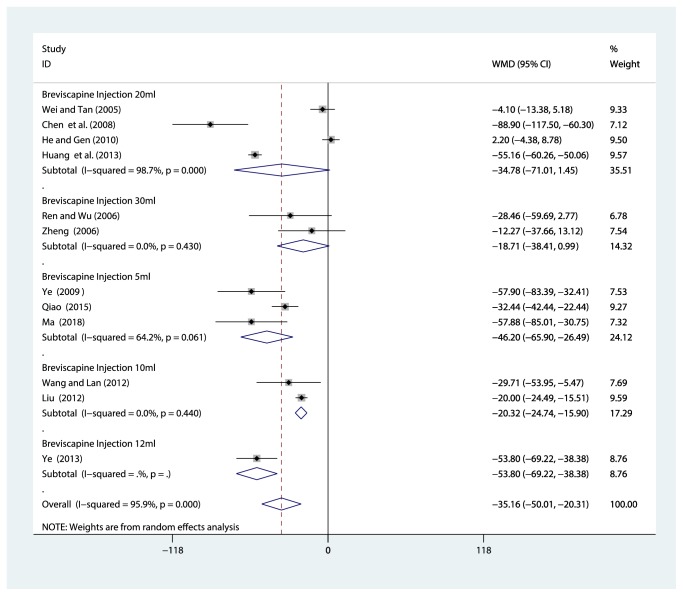
Subgroup analysis of different doses of breviscapine injection plus antihypertensive drugs versus antihypertensive drugs alone in terms of Scr for hypertensive nephropathy.

Because of variability in evaluating point of the serum creatinine, we conducted subgroup analysis among studies using different doses of breviscapine injection (30ml, 20 ml, 12 ml, 10 ml, and 5 ml). Compared with the control groups, the results of subgroup analysis showed that there was no significant correlation between the decrease of serum creatinine and the dose of breviscapine injection (Figure 6).

#### 3.4.4. 24-Hour Urinary Total Protein (24h UTP, g/d)

**Figure 7:**
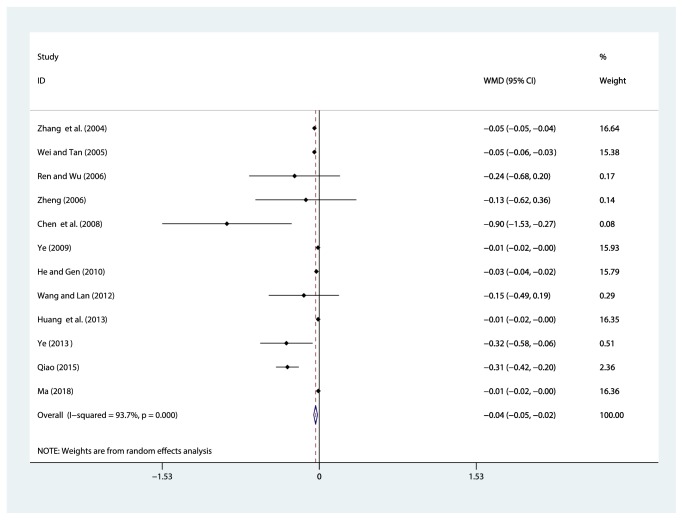
Meta-analysis results of breviscapine injection plus antihypertensive drugs versus antihypertensive drugs alone in terms of the 24 h UTP for hypertensive nephropathy.

Twelve studies [29–36, 38–40, 44] reported on the use of breviscapine injection plus antihypertensive drugs in terms of the 24 h UTP for hypertensive nephropathy. After the test for heterogeneity (*I*^2^ = 93.7%*, P* ≤ 0.001) (Figure 7), we employed a random effects model. We conducted a sensitivity analysis and applied Egger's test (*P* = 0.586) to evaluate publication bias for 24 h UTP (Supplementary Figures S4 and S5). The meta-analysis revealed that the experimental group performed better than the control group in reducing 24 h UTP [WMD = –0.04, 95% CI(–0.05, –0.02), *P* ≤ 0.001] (Figure S6).

#### 3.4.5. Blood Urea Nitrogen (BUN, mmol/L)

**Figure 8:**
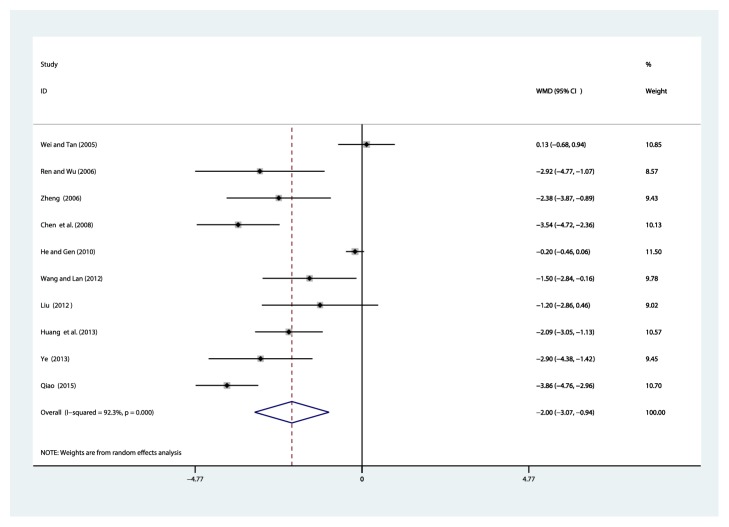
Meta-analysis results of breviscapine injection plus antihypertensive drugs versus antihypertensive drugs alone in terms of the BUN for hypertensive nephropathy.

Ten studies [30–33, 35–40] reported on the use of breviscapine injection plus antihypertensive drugs in the treatment of BUN for hypertensive nephropathy. After the test for heterogeneity (*I*^2^ = 92.3%, *P* ≤ 0.001) (Figure 8), we employed a random effects model. We conducted a sensitivity analysis and applied Egger's test (*P* = 0.015) to evaluate publication bias for BUN (Supplementary Figures S7 and S8). The meta-analysis revealed that the experimental group performed better than the control group in reducing BUN [WMD = –2.00, 95% CI(–3.07, –0.94), *P* ≤ 0.001] (Supplementary Figure S9).

#### 3.4.6. Creatinine Clearance Rate (Ccr, ml/min)

**Figure 9:**
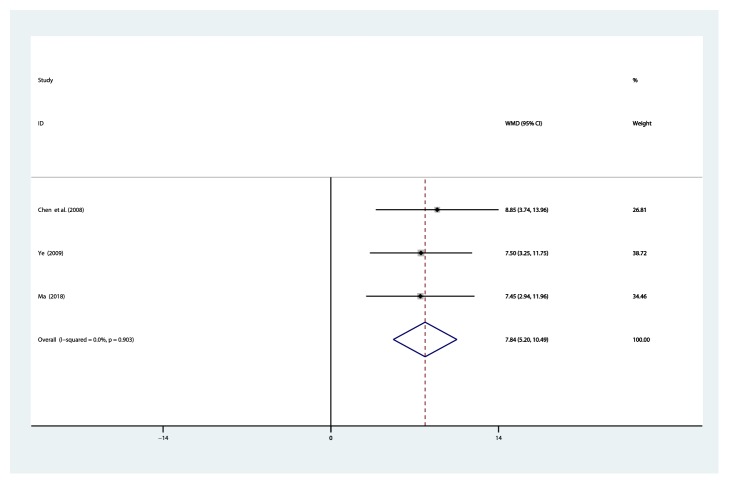
Meta-analysis results of breviscapine injection plus antihypertensive drugs versus antihypertensive drugs alone in terms of the Ccr for hypertensive nephropathy.

Three studies [33, 34, 44] reported on the use of breviscapine injection plus antihypertensive drugs in the treatment of Ccr for hypertensive nephropathy. After the test for heterogeneity (*I*^2^ = 0.0%, *P* = 0.903) (Figure 9), thus, the fixed-effects model was used for data analysis. The meta-analysis revealed that the experimental group performed better than the control group in improving creatinine clearance rate [WMD = 7.84, 95% CI(5.20, 10.49), *P* ≤ 0.001] (Figure S10).

#### 3.4.7. Beta-2-Microglobulin (B2M, mg/L)

**Figure 10:**
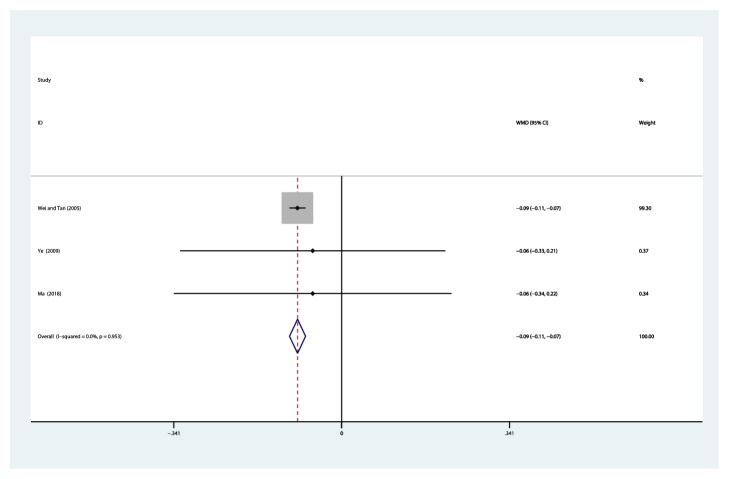
Meta-analysis results of breviscapine injection plus antihypertensive drugs versus antihypertensive drugs alone in terms of beta-2-microglobulin for hypertensive nephropathy.

Three studies [30, 34, 44] reported on the use of breviscapine injection plus antihypertensive drugs in the treatment of urine beta-2-microglobulin for hypertensive nephropathy. After the test for heterogeneity (*I*^2^ = 0.0%, *P* = 0.953) (Figure 10), thus, the fixed-effects model was used for data analysis. The meta-analysis revealed that the experimental group performed better than the control group in reducing B2M [WMD = –0.09, 95% CI(–0.11, –0.07), *P* ≤ 0.001] (Figure S11).

#### 3.4.8. Systolic Blood Pressure (SBP, mmHg)

**Figure 11:**
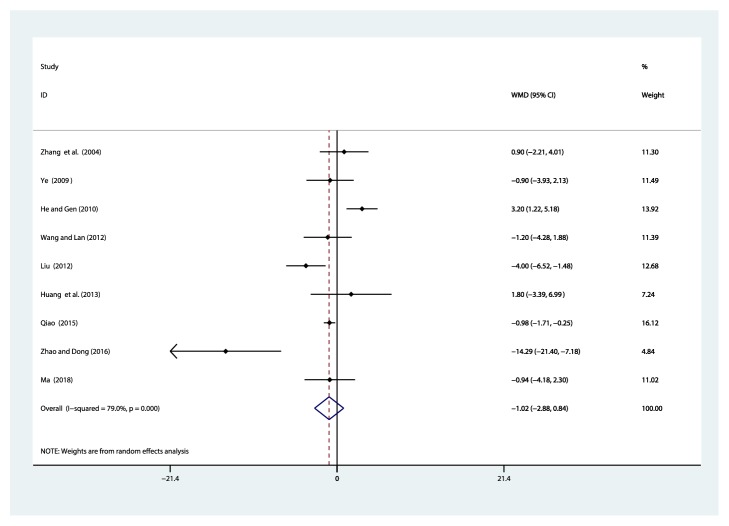
Meta-analysis results of breviscapine injection plus antihypertensive drugs versus antihypertensive drugs alone in terms of the SBP for hypertensive nephropathy.

Nine studies [29, 34–38, 40, 41, 44] reported on the use of breviscapine injection plus antihypertensive drugs in the treatment of SBP for hypertensive nephropathy. After the test for heterogeneity (*I*^2^ = 79%, *P* ≤ 0.001) (Figure 11), we employed a random effects model. Meta-analysis showed a nonsignificant trend for reduction in systolic blood pressure between the experimental group and the control group [WMD = –1.02, 95% CI(–2.88, 0.84), *P* = 0.281] (Figure S12).

#### 3.4.9. Comparison of Clinical Efficacy

**Figure 12:**
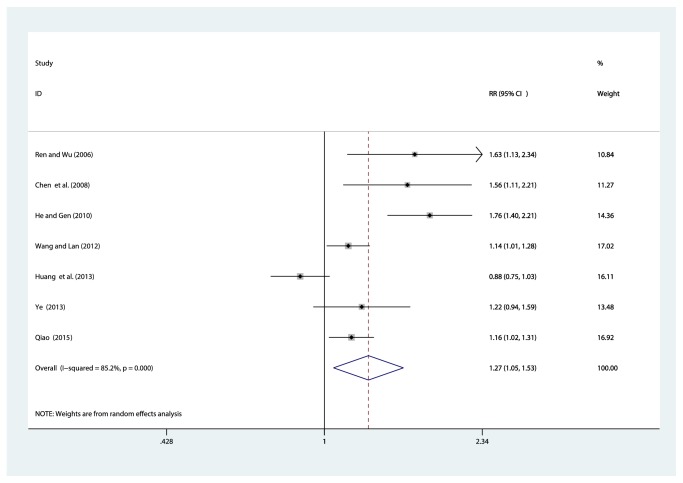
Meta-analysis results of breviscapine injection plus antihypertensive drugs versus antihypertensive drugs alone in terms of clinical efficacy for hypertensive nephropathy.

A total of 7 studies [31, 33, 35, 36, 38–40] reported the results of the total effective rate, involving 657 patients (330 in the experimental group and 327 in the control group). After the test for heterogeneity (*I*^2^ = 85.2%, *P* ≤ 0.001) (Figure 12), we employed a random effects model. We conducted a sensitivity analysis and applied Egger's test (*P* = 0.181) to evaluate publication bias for effective rate (Supplementary Figures S13 and S14). Meta-analysis indicated that the total clinical effective rate of breviscapine injection plus antihypertensive drugs versus antihypertensive drugs alone in treating for hypertensive nephropathy was higher than that in the control group, which showed a statistically significant difference [RR = 1.27, 95% CI(1.05, 1.53), *P* = 0.014] (Figure S15). All included trials were published in Chinese academic journals. Since trials with negative or neutral results are less likely to be published, the efficacy of published studies might be overestimated. Consequently, the possibility of publication bias could not be ruled out.

#### 3.4.10. Adverse Effects

Nine of the included trials [29–36, 38] described adverse effects in detail, while the others did not mention adverse events. Only one showed mild facial flushing during intravenous dripping of breviscapine [30], and after the speed of transfusion was slowed down, the symptom got remitted. Two cases [38] showed redness of limb skin in the experimental group. There were two cases [31, 32] of head inflation during the use of antihypertensive drugs. There were three cases [33, 34] of head swelling and dizziness during infusion during the use of breviscapine injection. Twenty-one cases showed dry cough due to the use of ACEI in six trials [29, 31–33, 35, 36]. None of the adverse events were serious. These symptoms are self-limited and did not affect treatment.

## 4. Discussion

This meta-analysis included 14 studies with 1,170 total participants comparing breviscapine injection plus antihypertensive drugs versus antihypertensive drugs alone for hypertensive nephropathy. Meanwhile, the results demonstrated that the expression levels of SCr and BUN were significantly lower in patients treated with breviscapine injection in comparison with control subjects, and the improvement of creatinine clearance was higher in patients treated with breviscapine in comparison with control subjects, suggesting that the drug serves as a protective role in the renal system of patients with hypertensive nephropathy. Microalbuminuria is regarded as the earliest clinical sign of hypertensive nephropathy. It is defined as a urinary albumin excretion rate ranging from 30 to 300 mg/day, and the definitive measurement is based on a timed urine collection during a 24-h period. The present meta-analysis indicated that breviscapine injection can reduce 24-hour urinary protein and the urinary beta-2-microglobulin; a reduction in urinary protein may contribute towards the renal protective effect of breviscapine injection in patients with hypertensive nephropathy. Our analysis revealed that experimental groups showed better overall clinical efficacy than control groups.

Erigeron breviscapus is a kind of traditional Chinese medicine. It was first recorded in the book of “Yunnan Materia Medica”. According to Chinese medicine theory, erigeron breviscapus is cold-natured, sweet, bitter, and pungent in taste, with the function of clearing heat, relieving toxicity, eliminating wind and dampness, activating blood and removing stasis, expediting channel and activating meridian, relieving inflammation, and alleviating pain. The monomer component of Chinese herbal medicine (CHM), also known as the natural pure compound drug, has recently attracted much attention. The natural extract artemisinin and its derivatives are good examples of monomer components of CHM that can treat diseases through various activities and can be a good starting point to uncover the mechanism of traditional Chinese medicine.

Hypertensive renal injury is major target-organ damage due to sustained high BP. Long-term hypertension can cause renal sclerosis and gradually progress to chronic renal failure. Positive control of hypertension is the key to preventing hypertensive renal damage. According to Chinese medicine theory, hypertensive renal injury is strongly related to fluid, phlegm, and dampness retention syndrome and liver-yang hyperactivity syndrome, which are caused by deficiency syndrome. Chinese medicines, which are used to treat fluid, phlegm, and dampness retention syndrome, deficiency syndrome, and liver-yang hyperactivity syndrome, respectively, have certain advantages with regard to treating hypertensive renal injury [45]. Clinical research indicates that Chinese herbal medicine might control increased SBP, inhibit the glomerular and tubular hyperplasia caused by high BP, and significantly reduce urinary albumin and beta-2-microglobulin by increasing the activity of renal rennin and the level of Ang II [46, 47].

## 5. Limitations

Several limiting factors in this study should be considered. First, the quality of the included randomized controlled trials was low. All included trials showed high or undefined risk of bias due to design, reporting, and methodology. There were no definitive, randomized, double-blind, placebo-controlled trials included in this meta-analysis. No trials reported detailed randomization methods or allocation concealment. No trial was double-blinded, and unfortunately, none of RCT (randomized controlled trial) has a placebo in the trial. Therefore, the reported strength of evidence should be reevaluated, and more rigorously designed, placebo-controlled trials are warranted to give high-level evidence in future studies [48]. Second, five trials did not report adverse reactions. Therefore, conclusions about safety cannot be made with confidence. Furthermore, certain active ingredients are chemically unstable, which limits large-scale synthesis. These pressing issues should be resolved in future research. The safety of breviscapine injection needs to be strictly monitored and properly reported in future clinical trials. Third, all tests produced positive results, although most tests were conducted with small samples. We tried our best to avoid language bias and positional prejudice, but cannot rule out potential publication bias.

Overall, our results suggest that breviscapine injection is effective and safe for the treatment of hypertensive nephropathy, and this work has reference value for clinicians. More large-scale, multicenter, rigorously designed, randomized, controlled trials are needed to provide accurate data to further validate the effectiveness and safety of breviscapine injection.

## 6. Conclusions

Evidence from this systematic review shows that breviscapine injection in combination with antihypertensive drugs can improve clinical efficacy and creatinine clearance rate and reduce serum creatinine, blood urea nitrogen, 24-hour urinary protein, and beta-2-microglobulin in hypertensive nephropathy patients. There is no evidence that breviscapine injection in combination with antihypertensive drugs can improve SBP in hypertensive nephropathy patients.

## Figures and Tables

**Table 1:** Characteristics of 14 studies fulfilling the inclusion criteria.

Study	Sample size (T/C)	Sex M/F	Age (years) range, mean	Diagnosis standards	Intervention	Control	Course of treatment	Clinical standards	Outcomes	Adverse reactions
Zhang et al. 2004[29]	94(47/47)	T: 18/16, C: 17/13	T: 60±8, C: 60±8	GMY (1999WHO-ISH)	Breviscapine injection (20 ml, ivgtt, qd) + control	Lisinopril (5-10 mg, po, tid), 25 mg, tid	2 weeks	Guiding principles (2002)	24UAER, SBP, DBP, B2M, TC, TG, LDL, LDH -C, Hct, PAGT, Fib	T: 2 cases of dry cough C: 3 cases of dry cough

Wei and Tan 2005[30]	76(40/36)	T: 30/10, C: 28/8	T: 63.5, C: 64.3	GMY (1999WHO-ISH)	Breviscapine injection (20 ml, ivgtt, qd) + control	Amlodipine (5 mg, po, qd)+ Captopril (25 mg, po, tid)	4 weeks	Guiding principles (2002)	24 h UTP, BUN, Scr, blood B2M, Urine B2M	T: 1 showed mild facial flushing during infusion C: None

Ren and Wu 2006[31]	60(30/30)	T: 21/9, C: 22/8	T: 44-75, C: 43-74	CGMY (2004)	Breviscapine injection (30 ml, ivgtt, qd) + control	Captopril (25-75 mg, po, qd) or Nifedipine (20-60 mg, po, qd)	4 weeks	Guiding principles (2002)	Clinical efficacy, 24 h UTP, BUN, Scr,	T: 2 cases of dry cough C: 1 case of mild head inflation

Zheng 2006[32]	72(37/35)	T: 22/13, C: 23/14	T: 41-73, C: 42-71	GMY (1999WHO-ISH)	Breviscapine injection (30 ml, ivgtt, qd) + control	Captopril (25-75 mg, po, bid or tid) or Nifedipine (10-60 mg, po, bid or tid)	4 weeks	Guiding principles (2002)	24 h UTP, BUN, Scr,	T: 2 cases of cough C: 1 case of mild head inflation

Chen et al. 2008[33]	57(28/29)	T: 19/9, C: 20/9	T: 45.6±20.3, C: 46.4±21.1	CGMY (2004)	Breviscapine injection (20 ml, ivgtt, qd) + control	Benazepril (no details)	4 weeks	Guiding principles (2002)	Clinical efficacy, 24 h UTP, BUN, Scr,, Ccr	T: 2 cases of head swelling, dizziness during infusion C: 1 case of cough

Ye 2009[34]	75(40/35)	T: 24/16, C: 20/15	T: 47.3±18.2, C: 46.9±16.1	GMY (1999WHO-ISH) and CGMY (2005)	Breviscapine injection (5 ml, ivgtt, qd) + control	Losartan Potassium (100 mg, po, qd)	4 weeks	Guiding principles (2002)	24 h UTP, Scr,, Ccr, Urine B2M, SBP, DBP	T: 1 case of dizziness C: None

He and Gen 2010[35]	168(84/84)	T: 49/35, C: 48/36	T: 69±11, C: 68±11	CGMY (2005)	Breviscapine injection (20 ml, ivgtt, qd) + control	Captopril (12.5-50 mg, po, tid)	30 days	Guiding principles (2002)	Clinical efficacy, SBP, DBP, 24 h UTP, Scr, BUN	T: 5 cases of dry cough C: 3 cases of dry cough

Wang and Lan 2012[36]	103(52/51)	Unclear	T: 62.4±4.8, C: 62.4±4.8	GMY (1999WHO-ISH) and Nephrology (Haiyan Wang)	Breviscapine injection (10 ml, ivgtt, qd) + control	Lisinopril (20 mg, po, qd)	30 days	Guiding principles (2002)	Clinical efficacy, SBP, DBP, 24 h UTP, Scr, BUN	T: 1 case of dry cough C: 2 cases of dry cough

Liu 2012[37]	50(26/24)	T: 12/14, C: 10/14	T: 55±4, C: 53±5	GMY (1999WHO-ISH) and CGMY (2005)	Breviscapine injection (10 ml, ivgtt, qd) + control	Benazepril (10 mg, po, bid)	2 weeks	Guiding principles (2002)	SBP, DBP, Urinary microalbumin	Not reported

Huang et al. 2013[38]	63(33/30)	T: 24/9, C: 19/11	Unclear	CGMY (2004)	Breviscapine injection (20 ml, ivgtt, qd) + control	Felodipine (5 mg, po, qd)+ Aspirin (100 mg, po, qd)	4 weeks	Guiding principles (2002)	Clinical efficacy, SBP, DBP, 24 h UTP, Scr, BUN, TC, TG, blood glucose	T: 2 cases of limb skin redness C: None

Ye 2013[39]	48(24/24)	T: 16/8, C: 14/10	T: 53.0±9.3, C: 49.0±11.6	CGMY (2010)	Breviscapine injection (12 ml, ivgtt, qd) + control	Antihypertensive Drugs (no details) + Prostaglandin E injection (2 ml, ivgtt, qd)	30 days	Guiding principles (2002)	Clinical efficacy, 24 h UTP, TC, TG, Scr, BUN,	Not reported

Qiao 2015[40]	158(79/79)	Unclear	T: 48.01±3.15, C: 48.01±3.15	GMY (1999WHO-ISH) and CGMY (2005)	Breviscapine injection (5 ml, ivgtt, qd) + control	Captopril (25-75 mg, po, tid) or Nifedipine (10-60 mg, po, tid)	4 weeks	Guiding principles (2002)	Clinical efficacy, SBP, DBP, 224 h UTP, Scr, BUN	Not reported

Zhao and Dong 2016[41]	80(40/40)	Unclear	T: 52.5±7.1, C: 52.5±7.1	CGMY (2010)	Breviscapine injection (5 ml, ivgtt, qd) + control	Benazepril (5mg, po, qd)	4 weeks	Guiding principles (2002)	Col-IV, LN, P III P, ET-1, MMP-9, NO, EILA, EISA, VOI, SBP, DBP	None

Ma 2018[41]	66(33/33)	T: 19/14, C: 20/13	T: 48.00±3.14, C: 48.99±2.98	GMY (1999WHO-ISH) and CGMY (2005)	Breviscapine injection (5 ml, ivgtt, qd) + control	Losartan Potassium (100mg, po, qd)	4 weeks	Guiding principles (2002)	Urine B2M, SBP, DBP, 24 h UTP, Scr, Ccr	Not reported

T: treatment group; C: control group; GMY (1999 WHO-ISH): The 1999 WHO-ISH Guidelines for the Management of Hypertension; CGMY (2004): 2004 Chinese Guidelines for the Management of Hypertension; CGMY (2010): 2010 Chinese Guidelines for the Management of Hypertension; CGMY (2014): 2014 Chinese Guidelines for the Management of Hypertension; Guiding Principles (2002): Guiding Principles of Clinical Research on New Drugs of Chinese Medicines (2002); Scr: serum creatinine; BUN: blood urea nitrogen; Ccr: creatinine clearance rate; 24 h UTP: 24-hour urinary total protein; B2M: beta-2-microglobulin; SBP: systolic blood pressure; DBP: diastolic blood pressure; TC: total cholesterol; TG: total triglycerides. PAGT: platelet aggregation rate; Fib: fibrinogen; Hct: hematocrit; LDL: low-density lipoprotein; LDH-C: lactate dehydrogenase C; Col-IV: collagen IV, LN: laminin; P III P: procollagen-III-peptide; ET-1: endothelin 1; MMP-9: matrix metalloproteinase-9; EILA: elasticity index of large artery; EISA: elasticity index of small artery; VOI: vascular overload index.

## Supplementary Materials

Supplementary Materials Supplementary Tables S1 and S2: search strategy. Supplementary Figure S1: Egger's test to evaluate publication bias of Scr. Supplementary Figure S2: meta-analysis results of breviscapine injection plus antihypertensive drugs versus antihypertensive drugs alone in terms of the Scr for hypertensive nephropathy. Supplementary Figure S3: sensitivity analysis for Scr. Supplementary Figure S4: sensitivity analysis for 24-hour urinary total protein. Supplementary Figure S5: Egger's test to evaluate publication bias of 24-hour urinary total protein. Supplementary Figure S6: meta-analysis results of breviscapine injection plus antihypertensive drugs versus antihypertensive drugs alone in terms of the 24 h UTP for hypertensive nephropathy. Supplementary Figure S7: sensitivity analysis for BUN. Supplementary Figure S8: Egger's test to evaluate publication bias of BUN. Supplementary Figure S9: meta-analysis results of breviscapine injection plus antihypertensive drugs versus antihypertensive drugs alone in terms of the BUN for hypertensive nephropathy. Supplementary Figure S10: meta-analysis results of breviscapine injection plus antihypertensive drugs versus antihypertensive drugs alone in terms of the Ccr for hypertensive nephropathy. Supplementary Figure S11: meta-analysis results of breviscapine injection plus antihypertensive drugs versus antihypertensive drugs alone in terms of the beta-2-microglobulin for hypertensive nephropathy. Supplementary Figure S12: meta-analysis results of breviscapine injection plus antihypertensive drugs versus antihypertensive drugs alone in terms of the systolic blood pressure for hypertensive nephropathy. Supplementary Figure S13: sensitivity analysis for clinical efficacy. Supplementary Figure S14: Egger's test to evaluate publication bias of clinical efficacy. Supplementary Figure S15: meta-analysis results of breviscapine injection plus antihypertensive drugs versus antihypertensive drugs alone in terms of clinical efficacy for hypertensive nephropathy.
