# Supplementary material for: Combined Therapy of Hypertensive Nephropathy with Breviscapine Injection and Antihypertensive Drugs: A Systematic Review and a Meta-Analysis
Source: Evid Based Complement Alternat Med. 2018 Dec 20;2018:2958717. doi: 10.1155/2018/2958717 (PMC6317107; doi:10.1155/2018/2958717)
Supplement: Supplementary Materials — Supplementary Tables S1 and S2: search strategy. Supplementary Figure S1: Egger's test to evaluate publication bias of Scr. Supplementary Figure S2: meta-analysis results of breviscapine injection plus antihypertensive drugs versus antihypertensive drugs alone in terms of the Scr for hypertensive nephropathy. Supplementary Figure S3: sensitivity analysis for Scr. Supplementary Figure S4: sensitivity analysis for 24-hour urinary total protein. Supplementary Figure S5: Egger's test to evaluate publication bias of 24-hour urinary total protein. Supplementary Figure S6: meta-analysis results of breviscapine injection plus antihypertensive drugs versus antihypertensive drugs alone in terms of the 24 h UTP for hypertensive nephropathy. Supplementary Figure S7: sensitivity analysis for BUN. Supplementary Figure S8: Egger's test to evaluate publication bias of BUN. Supplementary Figure S9: meta-analysis results of breviscapine injection plus antihypertensive drugs versus antihypertensive drugs alone in terms of the BUN for hypertensive nephropathy. Supplementary Figure S10: meta-analysis results of breviscapine injection plus antihypertensive drugs versus antihypertensive drugs alone in terms of the Ccr for hypertensive nephropathy. Supplementary Figure S11: meta-analysis results of breviscapine injection plus antihypertensive drugs versus antihypertensive drugs alone in terms of the beta-2-microglobulin for hypertensive nephropathy. Supplementary Figure S12: meta-analysis results of breviscapine injection plus antihypertensive drugs versus antihypertensive drugs alone in terms of the systolic blood pressure for hypertensive nephropathy. Supplementary Figure S13: sensitivity analysis for clinical efficacy. Supplementary Figure S14: Egger's test to evaluate publication bias of clinical efficacy. Supplementary Figure S15: meta-analysis results of breviscapine injection plus antihypertensive drugs versus antihypertensive drugs alone in terms of clinical efficacy for hypertensi [file 2958717.f1.docx]

**Supplementary Materials**

Supplementary Tables S1 and S2: search strategy.

Supplementary Figure S1: Egger’s test to evaluate publication bias of Scr.

Supplementary Figure S2: Meta-analysis results of breviscapine injection plus antihypertensive drugs versus antihypertensive drugs alone in terms of the Scr for hypertensive nephropathy.

Supplementary Figure S3: Sensitivity analysis for Scr.

Supplementary Figure S4: Sensitivity analysis for 24-hour urinary total protein.

Supplementary Figure S5: Egger’s test to evaluate publication bias of 24-hour urinary total protein.

Supplementary Figure S6: Meta-analysis results of breviscapine injection plus antihypertensive drugs versus antihypertensive drugs alone in terms of the 24 h UTP for hypertensive nephropathy.

Supplementary Figure S7: Sensitivity analysis for BUN.

Supplementary Figure S8: Egger’s test to evaluate publication bias of BUN.

Supplementary Figure S9: Meta-analysis results of breviscapine injection plus antihypertensive drugs versus antihypertensive drugs alone in terms of the BUN for hypertensive nephropathy.

Supplementary Figure S10: Meta-analysis results of breviscapine injection plus antihypertensive drugs versus antihypertensive drugs alone in terms of the Ccr for hypertensive nephropathy.

Supplementary Figure S11: Meta-analysis results of breviscapine injection plus antihypertensive drugs versus antihypertensive drugs alone in terms of the beta-2-microglobulin for hypertensive nephropathy.

Supplementary Figure S12: Meta-analysis results of breviscapine injection plus antihypertensive drugs versus antihypertensive drugs alone in terms of the systolic blood pressure for hypertensive nephropathy.

Supplementary Figure S13: Sensitivity analysis for clinical efficacy.

Supplementary Figure S14: Egger’s test to evaluate publication bias of clinical efficacy.

Supplementary Figure S15: Meta-analysis results of breviscapine injection plus antihypertensive drugs versus antihypertensive drugs alone in terms of clinical efficacy for hypertensive nephropathy.

**Supplementary Tables S1 and S2: search strategy.**

TABLE S1: Search strategies used for PubMed and other English language databases were as follows:

| #1 | Hypertension, Renal [MeSH Terms] OR “Hypertension, Renovascular” [Title/Abstract] OR “Hypertensive Nephropathy” [Title/Abstract] OR “Hypertensive Kidney Lesion” [Title/Abstract] OR “Hypertensive Renal Damage” [Title/Abstract] |
| --- | --- |
| #2 | “Breviscapine” [Title/Abstract] OR “Breviscapine Injection” [Title/Abstract] OR “Dengzhanhua” [Title/Abstract] OR “Dengzhanhua Injection” [Title/Abstract] OR “BVP” [Title/Abstract] OR “BVP Injection” [Title/Abstract] |
| #3 | (“randomized controlled trial [pt]” OR “controlled clinical trial [pt]” OR “randomized [tiab] OR placebo [tiab]” OR “clinical trials as topic [mesh: noexp]” OR “randomly [tiab]” OR “trial [ti]”) NOT (animals [mh] NOT humans [mh]) |
| #4 | #1 AND #2 AND #3 |

TABLE S2: Search strategies used for CNKI and other Chinese language databases were as follows (search terms were used as free-text terms and translated into Chinese):

| #1 | “Gao Xue Ya Shen Yan” [MeSH Terms] OR “Gao Xue Ya Shen Bing” [Title/ Abstract] OR “Gao Xue Ya Shen Shun Hai” |
| --- | --- |
| #2 | “Deng Zhan Hua” [MeSH Terms] OR “Dengzhanhua Preparations” [Title/Abstract] OR “Dengzhanhua Zhusheye” [Title/Abstract] OR “Dengzhanhua Zhusheye” [Title/Abstract] OR “Zhusheyong Dengzhanhua” [Title/Abstract] |
| #3 | “Suiji Duizhao Shiyan” [Title/Abstract] OR “Duizhao Linchuang Shiyan” [Title/Abstract] OR “Suiji” [Title/Abstract] |
| #4 | #1 AND #2 AND #3 |


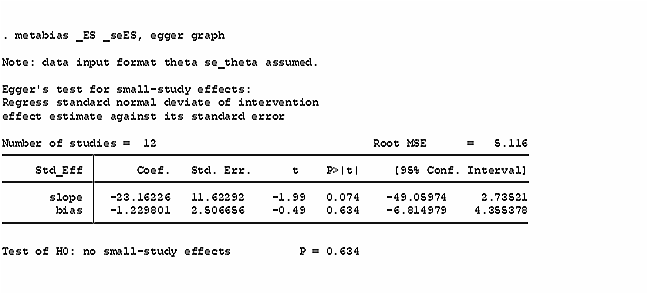


FIGURE S1: Egger’s test to evaluate publication bias of Scr.


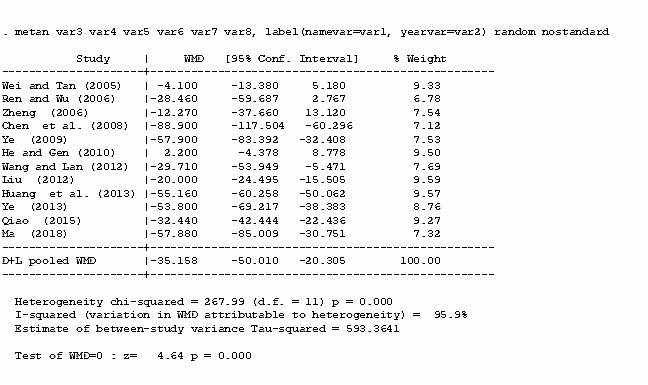


FIGURE S2: Meta-analysis results of breviscapine injection plus antihypertensive drugs versus antihypertensive drugs alone in terms of the Scr for hypertensive nephropathy.


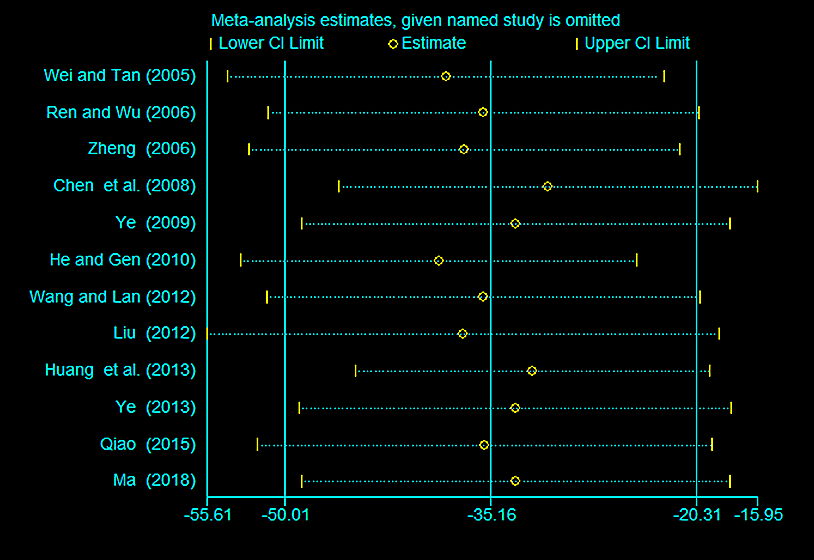


FIGURE S3: Sensitivity analysis for Scr.


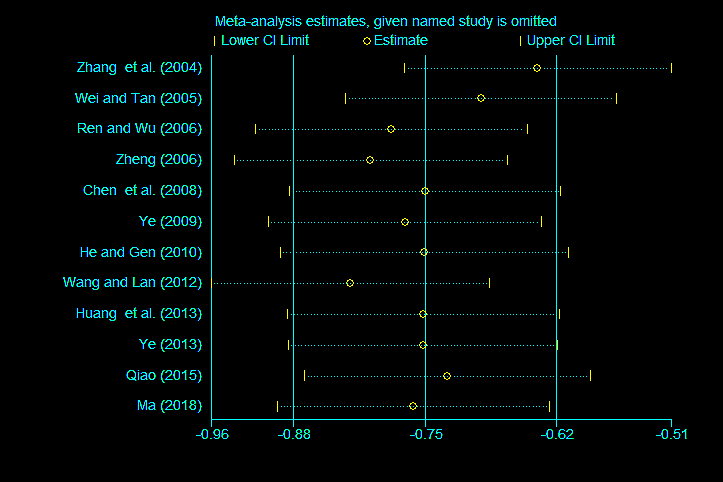


FIGURE S4: Sensitivity analysis for 24-hour urinary total protein


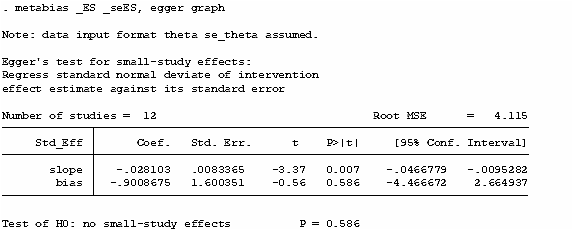


FIGURE S5: Egger’s test to evaluate publication bias of 24-hour urinary total protein.


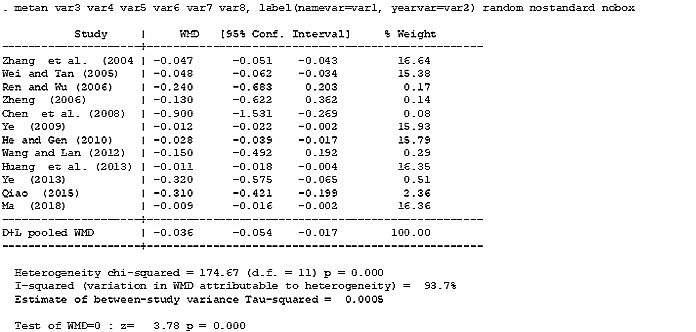


FIGURE S6: Meta-analysis results of breviscapine injection plus antihypertensive drugs versus antihypertensive drugs alone in terms of the 24 h UTP for hypertensive nephropathy.


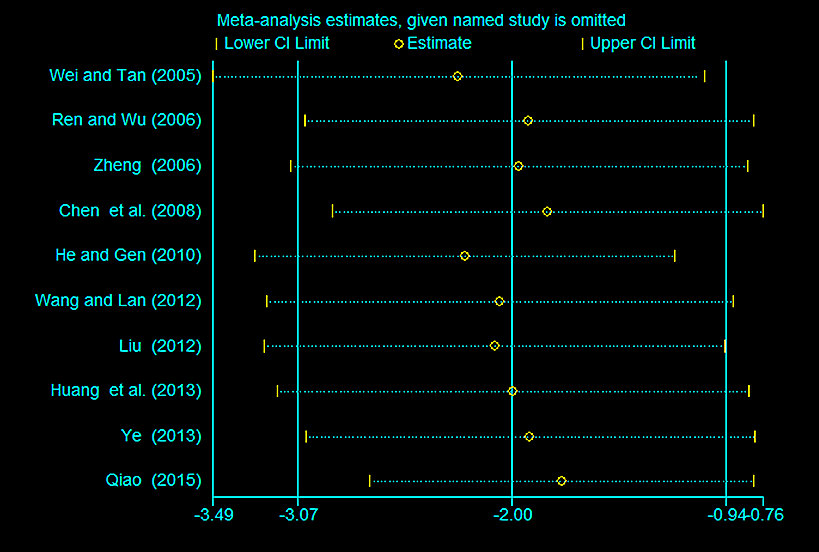


FIGURE S7: Sensitivity analysis for BUN.


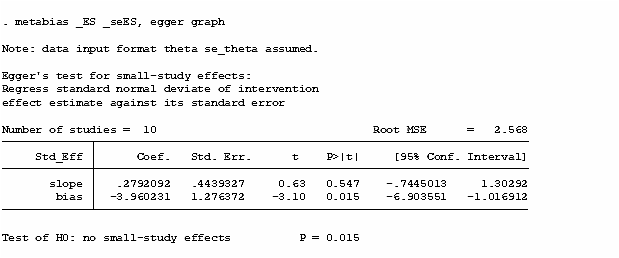


FIGURE S8: Egger’s test to evaluate publication bias of BUN.


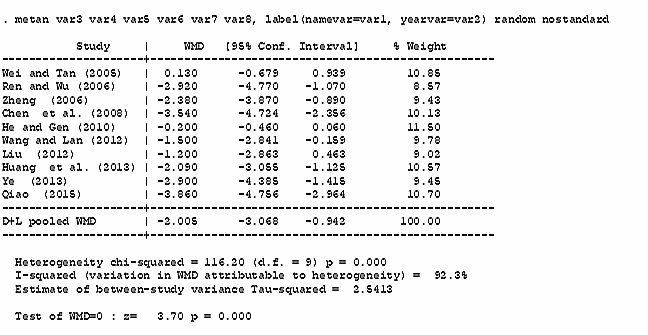


FIGURE S9: Meta-analysis results of breviscapine injection plus antihypertensive drugs versus antihypertensive drugs alone in terms of the BUN for hypertensive nephropathy.


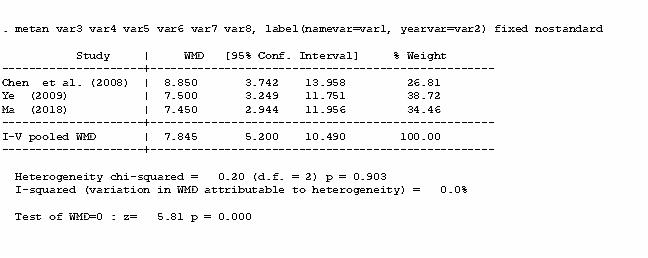


FIGURE S10: Meta-analysis results of breviscapine injection plus antihypertensive drugs versus antihypertensive drugs alone in terms of the Ccr for hypertensive nephropathy.


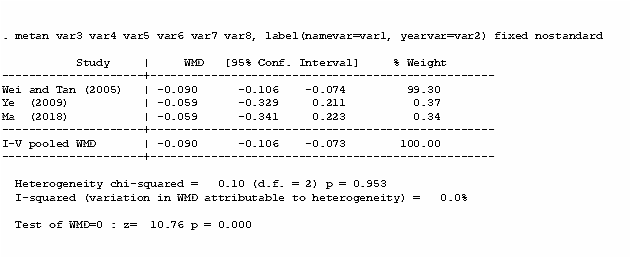


FIGURE S11: Meta-analysis results of breviscapine injection plus antihypertensive drugs versus antihypertensive drugs alone in terms of the beta-2-microglobulin for hypertensive nephropathy.


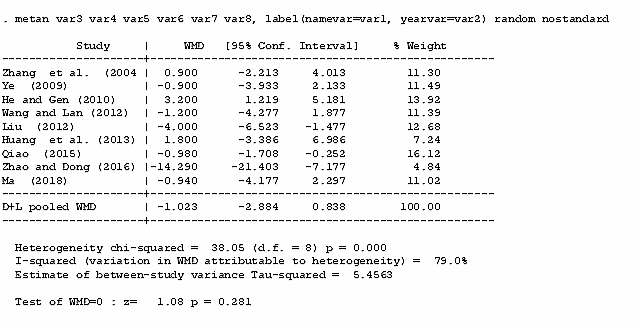


FIGURE S12: Meta-analysis results of breviscapine injection plus antihypertensive drugs versus antihypertensive drugs alone in terms of the systolic blood pressure for hypertensive nephropathy.


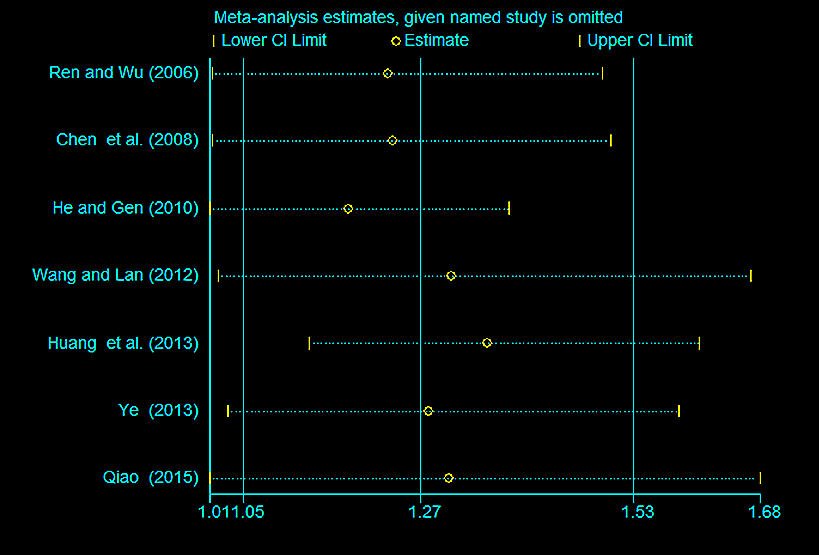


FIGURE S13: Sensitivity analysis for clinical efficacy.


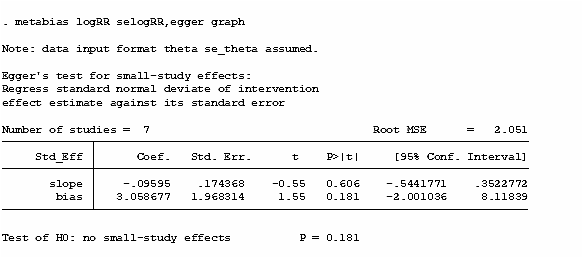


FIGURE S14: Egger’s test to evaluate publication bias of clinical efficacy.


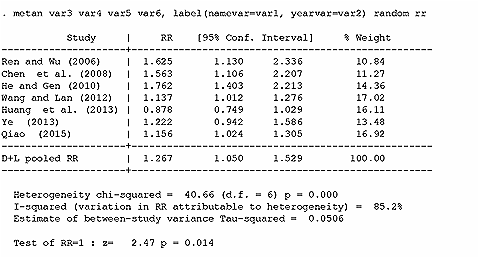


FIGURE S15: Meta-analysis results of breviscapine injection plus antihypertensive drugs versus antihypertensive drugs alone in terms of clinical efficacy for hypertensive nephropathy.
